# Compilation of climate data from heterogeneous networks across the Hawaiian Islands

**DOI:** 10.1038/sdata.2018.12

**Published:** 2018-02-13

**Authors:** Ryan J. Longman, Thomas W. Giambelluca, Michael A. Nullet, Abby G. Frazier, Kevin Kodama, Shelley D. Crausbay, Paul D. Krushelnycky, Susan Cordell, Martyn P. Clark, Andy J. Newman, Jeffrey R. Arnold

**Affiliations:** 1 University of Hawai‘i at Mānoa, Department of Geography, Honolulu, Hawai‘i 96822, USA; 2 USDA Forest Service, Institute of Pacific Islands Forestry, Hilo, Hawai‘i 96720, USA; 3 National Oceanic and Atmospheric Administration, National Weather Service, Honolulu, Hawai‘i 96822, USA; 4 Conservation Science Partners, Fort Collins, Colorado 80528, USA; 5 University of Hawai‘i at Mānoa, Department of Plant & Environmental Protection Sciences, Honolulu, Hawai‘i 96822, USA; 6 National Center for Atmospheric Research, Boulder, Colorado 80305, USA; 7 United States Army Corps of Engineers, Climate Preparedness and Resilience Program, Seattle, Washington 98124, USA

**Keywords:** Atmospheric dynamics, Hydrology, Environmental chemistry

## Abstract

Long-term, accurate observations of atmospheric phenomena are essential for a myriad of applications, including historic and future climate assessments, resource management, and infrastructure planning. In Hawai‘i, climate data are available from individual researchers, local, State, and Federal agencies, and from large electronic repositories such as the National Centers for Environmental Information (NCEI). Researchers attempting to make use of available data are faced with a series of challenges that include: (1) identifying potential data sources; (2) acquiring data; (3) establishing data quality assurance and quality control (QA/QC) protocols; and (4) implementing robust gap filling techniques. This paper addresses these challenges by providing: (1) a summary of the available climate data in Hawai‘i including a detailed description of the various meteorological observation networks and data accessibility, and (2) a quality controlled meteorological dataset across the Hawaiian Islands for the 25-year period 1990-2014. The dataset draws on observations from 471 climate stations and includes rainfall, maximum and minimum surface air temperature, relative humidity, wind speed, downward shortwave and longwave radiation data.

## Background & Summary

**Figure 1:**
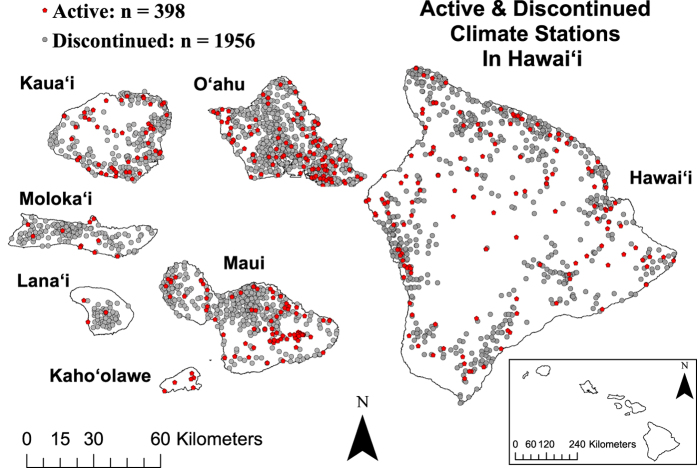
Active (as of 1/1/2017) and discontinued raingauge stations in Hawai‘i.

Many research endeavours are limited by lack of information on data availability. While a remarkable number of climate observations have been taken in the Hawaiian Islands, a comprehensive list of monitoring stations has been lacking, and hence many research projects might be unnecessarily data starved. Since 1838, at least 2,354 climate monitoring stations have operated in Hawai‘i, and 398 of these stations were active at the start of 2017 (Figure 1). The majority of the climate stations in the State have exclusively measured daily rainfall; however, some stations have also reported minimum and maximum temperature and other variables (Table 1). Precipitation in Hawai‘i consists of rainfall, different types of frozen precipitation (e.g., snow, sleet, hail, and freezing rain), and fog drip^1^. Considering that frozen precipitation is a minor overall contributor and fog drip is not accounted for in the data presented here, the term rainfall is used throughout the paper.

During the past several decades, efforts in Hawai‘i have focused on measuring multiple climate variables at fine temporal resolutions. In the late 1980s, measurements of additional climatic variables including relative humidity and radiative fluxes across elevation gradients became available with the establishment of the Haleakalā Climate Network (HaleNet) on the leeward slopes of Haleakalā a dormant volcano comprising the eastern portion of Maui Island^2^. HaleNet was the first permanent, advanced, climate network established in the state and is still in operation today. Other advanced climate networks exclusive to the State of Hawai‘i include the Hawai‘i Volcanoes Climate Network (HavoNet), the Hawai‘i Ecological Climate Observatory (HECO), the Haleakalā Crater Network (CraterNet)^2^ and the Little Haleakalā Climate Network (Little HaleNet)^2,3^. Other Hawai‘i-only networks that measure rainfall exclusively include the Hydrological Rainfall Network (Hydronet) and the Hawaiian Commercial and Sugar Company (HC&S) meteorological network.

Many climate stations in Hawai‘i are part of national or international networks. The Remote Automated Weather Station (RAWS) network, the United States Climate Reference Network (USCRN)^4^, and the Automated Surface Observing System (ASOS_AWOS) are all national networks. Federal organizations such as the National Renewable Energy Laboratory (NREL) and the United States Geological Survey (USGS) operate climate and water resource monitoring stations nationally. Much of the historical rainfall and temperature data in Hawai‘i comes from the National Weather Service (NWS) Cooperative Observer Program (COOP) network and the Community Collaborative Rain, Hail, & Snow Network (CoCoRaHs), which are both recognized as international networks. In this work, international networks are defined as, networks that are run and maintained by US agencies but include stations located outside of the US. Hawai‘i is also home to Mauna Loa Observatory (MLO), one of the six atmospheric baseline observatories within NOAA’s Earth System Research Laboratory’s global monitoring division (ESRL_GMD) from which numerous in-situ atmospheric measurements are conducted^5^. All of the climate stations identified in this work are part of the above mentioned networks.

A large number of climate measurements have been made at locations throughout Hawai‘i. However, the tasks of identifying the various data sources, acquiring the data (including data rescue), and developing consistent protocols for data standardization are not straightforward. The stations within different networks measure the same atmospheric variables, but inhomogeneities between networks and among individual stations require implementing appropriate data management and analysis protocols. Inhomogeneities between networks can include differences in: time-of measurement, measurement output intervals, temporal aggregation methods, time-stamp formats, missing value formats, measurement units, QA/QC protocols, length of records, sensor heights, sensor and data logger quality, and calibration and maintenance protocols^2,6–12^. These differences can create artefacts that limit the utility of the data unless appropriate QA/QC protocols are implemented^13^. Data rescue involves organizing and preserving climate data at risk of being lost due to deterioration, destruction, neglect, and/or technical issues^14^. The World Meteorological Organization has put forth guidelines on the best practices for organizing and implementing data rescue efforts^14^.

Creating a homogeneous and robust dataset requires attention to as many of these issues as possible. Problems arising from issues such as calibration uncertainty^5,6^ and changes associated with instrument replacement^15^ might not be easily addressed due to a lack of historical metadata, experimental costs, or lack of manpower. Any study including information from different networks requires reformatting all data to common file formats and time-steps, using consistent codes, e.g., to define missing values, and implementing some level of consistent QC protocols to standardize the dataset^8^.

The purpose of this paper is to describe the availability of climate data in Hawai‘i from both active and discontinued stations, the methods used for data acquisition and standardization, and to provide access to a quality controlled multivariate data set for the 25-year period 1990–2014. More specifically this article discusses: (i) step-by-step method for data quality control, (ii), methods for gap filling rainfall and temperature time series, and (iii) a description of the unique climate networks in the State of Hawai‘i.

The year 1990 was selected as the start date for the datasets accompanying this manuscript due to the improved spatial coverage of stations, especially for the higher elevations, and the increased variety of measured variables, i.e., relative humidity, wind speed, and shortwave and longwave radiation, relative to prior years. For rainfall, a complete dataset of monthly totals for the period 1920–2012 is available through the Rainfall Atlas of Hawai‘i website^1^. A comparable dataset for average monthly temperature is currently in development.

## Methods

### Data compilation

#### Hawai‘i-only networks

Monitoring the climate in Hawai‘i is not a trivial task. Many important climate stations in Hawai‘i are found in remote locations. Most of the stations in the HaleNet, Little HaleNet, CraterNet, HavoNet, and HECO networks, for example, can be accessed only by hiking into remote areas (see Data Records section for individual network descriptions). Some stations, such as those in the Little HaleNet network require a ~26-km (16-mi) hike through the upper reaches of Haleakalā National Park on Maui to access. For some remote high elevation HaleNet sites, access by helicopter is the only option.

To compile a comprehensive Hawai‘i-wide dataset, the first step is to identify and acquire data from all relevant climate networks. The data measured in Hawai‘i-only networks are typically not available in open access electronic repositories. Data from these networks generally can be obtained only from project principal investigators (PI’s) or data managers. Climate data from the HaleNet, Little HaleNet, CraterNet, HavoNet, and HECO networks are managed by the Giambelluca Ecohydrology Laboratory at the University of Hawai‘i at Mānoa. Data acquired from the HC&S and HydroNet networks were obtained by directly contacting the respective data managers. In one instance, data were rescued via floppy disc from a ~30-year-old personal computer at the HC&S facility on Maui.

Manually read stations are still in widespread use in Hawai‘i, requiring digitization of paper records. Partly because of the need to digitize these data, a substantial lag exists between the time of observation and the time data are made publically available on electronic repositories such as the NCEI. The office of the Hawai‘i State Climatologist serves as the repository for the paper records of climatic data. These data must be hand digitized from the paper records. To assure fidelity to the original written record, data are manually entered independently by two individuals and cross checked to detect entry errors.

#### National and international data networks

Much of the rainfall and temperature data compiled here were obtained from the Global Historical Climate Network (GHCN). The GHCN is a global database managed by the National Oceanic and Atmospheric Administration (NOAA) and hosted on the NCEI electronic data repository. Data from the ESRL/GMD, RAWS, USCRN, ASOS_AWOS, COOP and CoCoRaHs networks are all included in GHCN database (see Network Metadata section below for individual network descriptions).

NCEI is a useful resource for extracting GHCN data. However, this database does not always include all of the stations from a given network, all of the available data measured by an individual station, the highest temporal resolution of measurements at a station, or complete metadata for a station including the type of instrumentation used, instrument calibration and replacement records, and instrument measurement height and measurement protocols. In addition, data hosted by NCEI can sometimes disagree with the data obtained directly from an individual network source. For example, we found in this analysis that in some instances daily rainfall data from the RAWS network hosted by the NCEI was in disagreement with the hourly RAWS rainfall data (converted to daily) obtained from the Western Regional Climate Center (WRCC). The NCEI uses a multi-tiered QA approach that consists primarily of an automated procedure followed by occasional manual data record integrity checks^8,9^. Given the large number of station records and frequent additions of near-real-time and historical data, system-wide manual verification of QA algorithms is not possible^8^. When possible, the data used in the study were obtained directly from individual network sources rather than the NCEI archive. Data were also acquired at the highest temporal resolution available and then aggregated to the daily time step.

Discovery, acquisition, and compilation of Hawai‘i climate data for this study was extremely time consuming. Several person-months were needed to identify the available climate stations in the state, and it took well over a person-year to acquire the data and to compile a comprehensive list of stations. This is in addition to the time and effort invested by others in prior efforts that benefited this compilation.

### Quality assessment and quality control

Raw digital climate data are subject to a wide variety of errors, which can be introduced at any time during the data management chain of collection, processing, transfer and storage^11^. A broad set of recommended procedures for rescuing and quality controlling data have been developed by the World Meteorological Organization (WMO)^12,14,16^. Data acquired from various sources must be screened using a strict set of QA/QC protocols before any scientific analysis can be done. While some networks such as those included in the GHCN indicate that data have already been quality controlled^9^, all of the data provided here have undergone an additional set of QC that ensures a level of data homogeneity. When dealing with hourly- or sub-hourly-interval “raw” data files (files obtained directly from station data loggers) a set of pre-processing protocols are first applied to concatenate different files, eliminate duplicate records, and reformat the data. Once a common format is established the following set of QC protocols are applied. Measurement times are adjusted to an even output interval (e.g. 15 min data recorded at 10:18,10:33, 10:48 and 11:02 are set to the measurement times 10:15, 10:30, 10:45 and 11:00).

1. All time stamps are converted to local time (Hawaiian Standard Time (HST), UTC -10).
2. Time stamps are converted to a uniform format.
3. Missing values are replaced with NA.
4. All sub-hourly data are aggregated to an hourly interval.
5. Time stamps are adjusted to represent reporting time at the end of an hour.
6. All measurements are converted to metric units.
7. Basic screening for outliers is done using variable-specific thresholds applied to hourly data. Thresholds are set by observed historical variability, instrument limitations, and local knowledge about environmental phenomena. Note: these thresholds are specific to the Hawai‘i region as a whole and station-specific thresholds were not considered.
8. Hourly data are aggregated to daily data for each day with 24 hourly values available.
9. Climate stations reporting measurements at the daily time step are subject to a different set of QC protocols (see below).
10. All of the stations were plotted in ArcGIS 10.3^17^ to verify a land based location. Stations with unresolved geographical coordinates were removed from the dataset.

During this analysis, we found that the most efficient way to process the data was to tailor a QC algorithm to each unique network. In some instances, several algorithms were used to meet all of the QC protocols depending on the attributes of an individual network in question. While writing a universal QC algorithm that could be applied to all of the data was not a goal of this project, we hope that this current work will provide guidance for the development of a more sophisticated and universal QC algorithm in the future.

#### Quality control for hourly data

Below we provide a step-by-step approach for the quality control that was implemented on the hourly climate data.

1. Rainfall (RF)
2. Negative RF is set to 0.
3. RF above 120 mm/h is flagged.
4. All flags are manually investigated to confirm that a heavy RF event occurred. To accomplish this:
5. Hourly and or daily RF at a nearby station is examined to confirm a heavy RF event.
6. Local newspaper archives and/or monthly rainfall summaries distributed by the NWS are examined.
7. If a heavy RF event is not confirmed than these data points are set to missing.
8. For tipping-bucket rain gauge records, the beginning and end of each file are checked for intentional or inadvertent spurious tips that occur during station maintenance.
9. Surface Air Temperature (T_a_)
10. Average hourly T_a_ values outside the range −9 C to 38 °C are set to missing.
11. Relative Humidity (RH)
12. RH less than -2% is set to missing
13. RH between -2% and 0% is set to 0%.
14. RH between 100 to 106% is set to 100%.
15. RH exceeding 106% is set to missing.
16. Wind Speed (WS)
17. Hourly WS outside the range 0 to 49 m s^−1^ is set to missing.
18. Shortwave Downward Radiation (Sw)
19. Hourly Sw outside the range -10 to 1367 W m^−2^ is set to missing.
20. Hourly Sw between -10 to 0 W m^−2^ is set to 0
21. All night time Sw values are set to 0
22. Longwave Downward Radiation (Lw)
23. Lw outside±250 W m^−2^ is set to missing. Note: ±250 is a typical sensor measurement range of raw Lw radiation (relative to the sensor).
24. To calculate an actual Lw hourly value, the raw Lw measurement is corrected by accounting for the body temperature of the instrument using the following equation.

$$Lw_{h}=Lw_{raw}+\sigma T^{4}$$

where Lw_h_ is the hourly longwave radiation, Lw_raw_ is the sensor measurement, σ is the Stefan-Boltzmann constant (5.67*10^−8^), and *T* is the body temperature of the instrument (K).

#### Quality control for daily data

Below we provide a step-by-step approach for the quality control that was implemented on the daily climate data.

1. Rainfall
2. Daily rainfall was assigned to calendar days. For many networks, rainfall measurements are reported once per day with no information as to when rainfall actually occurred. For example, if a manual raingauge is read at 8:00 AM on a given day, any rainfall amount >0 could have occurred between the measurement time on a given day and the measurement time of the previous day. To address this issue, the following set of protocols was applied.
3. The daily measurement was divided by the number of hours between a measurement time on a given day and the measurement time on the previous day to produce an average hourly value.
4. Average hourly values are assigned to each calendar day.
5. Hourly values are then summed to produce a daily RF value.
6. Daily rainfall exceeding 500 mm d^−1^ is flagged.
7. All flags are manually investigated to confirm that a heavy RF event occurred. To accomplish this:
8. Hourly and or daily RF at a nearby station is examined to confirm a heavy RF event.
9. Local newspaper archives and/or monthly rainfall summaries distributed by the NWS are examined.
10. If a heavy RF event is not confirmed than these data points are set to missing.
11. Maximum (T_max_) and Minimum (T_min_) air temperature
12. Daily T_max_ values outside the range 0 to 38 °C are set to missing.
13. Daily T_min_ values outside the range −11 to 33 °C are set to missing.
14. If T_min_ > T_max_ both values are set to missing.

Range limits were established using several methods. The RF flag threshold was based on the near maximum value found at HaleNet station HN164. HN 164 is located at Big Bog, Maui which has recently been identified as the wettest place in Hawai‘i^1^. Flagged values were investigated by cross referencing, both co-located stations (when available) or nearby stations as well as historical weather reports archived by the NWS. For T_max_ and T_min_, thresholds were established based on the maximum and minimum near surface air temperature historical thresholds for the State of Hawai‘i established by NOAA. The RH range limits were established based on suggested instrument uncertainty thresholds established by Vaisala (Vantaa, Finland), for their HMT35C and HMT45C relative humidity sensors, the instrumentation used for many of the RH stations in Hawai‘i. For Sw, the approximate solar constant (1367 W m^−2^) is used as the maximum threshold. Maximum thresholds for Ws and Lw were established based on the observed maxima found in the HaleNet measurement records at high elevation stations within the network.

We acknowledge that the QC protocols described here are simple in nature and focus primarily on formatting the data, range limit testing, and internal consistency checks^12^. Temporal consistency tests, which test the variation of a variable over time, were not applied to the time series. Analytical or statistical methodologies can be used to establish the expected change in a particular variable at any time interval. This can be done but comparing the prior and subsequent observations with one in question and/or by examining the entire time series. Common techniques for analyzing time series are auto regression and moving average analyses^12^. Models can also be fit to the data to identify discontinuities over time e.g. ref. 15. Once time series data have been modeled by an appropriate function the relationship between the data and the model can be used to make assessments or adjustments to the data^12,15^. We refer data users to guidelines for completeness checks, data consistency testing, and time series analysis proposed by the WMO^12^.

Daily measurement times varied between individual networks as well as individual stations. In some cases, measurement times are given with the data. When the measurement time is not available, the most recent known daily measurement time is assigned to that measurement.

We caution users that measurements of the climate variables in the datasets provided have not been adjusted to a common reference height (e.g. 2 m). Instrument height for individual climate stations can either be obtained by contacting respective network observers and or data managers.

### Gap filling

### Rainfall

**Figure 2:**
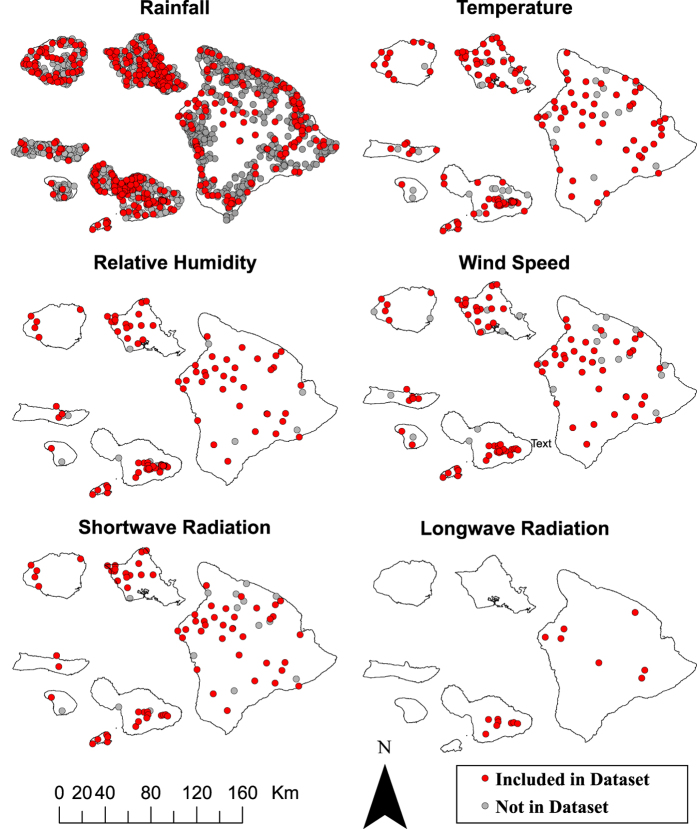
Spatial distribution of measured climate variables in Hawai‘i. Showing (red) stations included in the datasets accompanying this manuscript; and (gray) climate stations not included in the accompanying datasets.

Gap filling a RF time series allows for the enhancement of the spatial coverage of climate information. The number of active RF stations represents only 16.5% of the total number of rainfall gauge stations that have operated in the state. The RF dataset accompanying this manuscript includes data from 471 individual climate stations with at least three years of data over the 25-year period 1990-2014 (Figure 2). These data were used to fill as many gaps in the time series as possible using the normal ratio method^18^. This method uses the ratios of the mean of the station in question to the means of the most well correlated stations as adjustment factors in the estimation procedure. Gap filling using the normal ratio method has previously been used to fill monthly RF data in Hawai‘i^1^. The normal ratio gap filling method can be expressed with the following equation:

$$P_{x}=\frac{1}{n}\left(\sum_{i=1}^{n}P_{i}\frac{N_{x}}{N_{i}}\right)$$

Where, P_x_=predicted RF at station x, N_x_=average RF at station x, N_i_ =average RF at the ith-best correlated station, and P_i_=RF at the ith-best correlated station.

Following the methods of Giambelluca et al.^1^, the four stations with the highest correlation to a station in question (regardless of island) were identified as potential predictor stations. Next, a minimum correlation (r) of 0.88 was set for selection of predictor stations, and the ratios were computed for the overlapping period of data between stations. A minimum of 100 overlapping days was required. If the ratio was less than 0.3 or greater than 2.7 the predictor station was not used. This range of ratios was slightly greater than the one proposed by Giambelluca et al.^1^ (0.5 < ratio <2.5) to fill monthly RF data. The ratio range was expanded to provide more flexibility in the gap filing approach due to the greater variability that exists in a daily as opposed to a monthly RF dataset.

### Maximum and minimum temperate

Daily T_max_ and T_min_ datasets were gap-filled using linear regression. In total, data from 142 stations were used (Figure 2). Stations with a minimum correlation (r) of 0.71 (based on 100 comparisons) were used as predictor stations. For each station in question, a predictor station with the highest correlation was used to predict a missing value based on the linear relationship between the two stations. Daily gaps were filled on all of the days when an observation from a predictor station was available.

### Data Records

A list of data sources is included in Table 2. Individual datasets are available for download from the figshare electronic repository (Data Citation 1). These datasets include a 25-year time series for 7 climate variables (1990 to 2014) and a comprehensive list of discontinued and active climate stations in Hawai‘i. Below, we provide a detailed description of each individual climate network and information on data accessibility. Note: not all network data described below are included in the datasets accompanying this manuscript. Networks used for each of the 7 climate variable datasets accompanying this manuscript can be found in Table 2. Data were obtained in SI/Metric units unless otherwise noted.

### Network metadata

#### HaleNet—Hawai‘i-only network

The Haleakalā Climate network (HaleNet) consists of 11 full meteorological stations, eight of which are still in full operation and one is in partial operation (measuring RF only). HaleNet stations have been recording climatic data since June of 1988^2^, with rainfall recorded at a 1-minute interval since 1999. The data collected at these stations include measurements of net and solar radiation, T_a_, WS and wind direction(WD), RH, RF, soil temperature (T_s_), soil heat flux (SHF), and soil moisture (SM). More recent upgrades (2011) to the stations still in full operation have been the addition of 4-component net radiometers, which provide measurements of downward and upward longwave and shortwave radiation. These data are managed by the Giambelluca Ecohydrology Laboratory located in the Department of Geography at the University of Hawai‘i at Mānoa (https://sites.google.com/a/hawaii.edu/ecohydrology_lab/home).

#### HECO—Hawai‘i-only network

The Hawai‘i Ecological Climate Observatory (HECO) is a partnership between  the USDA Forest Service Pacific Southwest Research Station, Institute of Pacific Islands Forestry,  the University of Hawai‘i at Hilo, the University of Hawai‘i at Mānoa, and the University of California at Los Angeles. A total of eight HECO climate stations were established, all on Hawai‘i Island, seven of which are currently in operation. These stations have been collecting data since 2009 and include measurements of T_a_, WS and WD, RH, RF, T_s_, SHF, SM and both short and longwave radiation fluxes. Two of the HECO sites havestations are tower-mounted stations in forest ecosystems and four of the stations have been established to complement permanent vegetation plots associated with the Center for Tropical Forest Science permanent plot network (http://ctfs.si.edu/). Measurements are made at 10-min intervals. These data are managed jointly by The USDA Forest Service, Institute of Pacific Islands Forestry located in Hilo, Hawai’i (https://www.fs.fed.us/psw/programs/ipif/) and the Giambelluca Ecohydrology Laboratory, University of Hawai‘i at Mānoa (https://sites.google.com/a/hawaii.edu/ecohydrology_lab/home).

#### HavoNet—Hawai‘i-only network

Two field sites are located within Hawai‘i Volcanoes National Park (HavoNet) representing native forest and a forest invaded by an alien tree species, both within the cloud zone on the slopes of Kilauea Volcano on Hawai‘i Island. These sites are equipped with an extensive array of instruments measuring: RF, T_a_, RH, WS, WD, T_s_, SHF, SM, and downward and upward longwave and shortwave radiation fluxes. In addition, these sites are also equipped with eddy covariance sensors to monitor energy exchange and fluxes of water and carbon between the atmosphere and the ecosystem. Eddy covariance measurements are taken at 10-Hz, RF at a 1-minute interval, and all other climate measurements are recorded at 30 min intervals. These data are managed by the Giambelluca Ecohydrology Laboratory, University of Hawai‘i at Mānoa (https://sites.google.com/a/hawaii.edu/ecohydrology_lab/home).

#### ESRL/GMD—International Network

Mauna Loa Observatory (MLO) is a premier atmospheric research facility that has been continuously monitoring and collecting data related to atmospheric change since the 1950's. The undisturbed air, remote location, and minimal influences of vegetation and human activity at MLO are ideal for monitoring changing atmospheric conditions. The observatory is managed by the National Oceanic and Atmospheric Administration (NOAA) - Earth System Research Laboratory (ESRL) - Global Monitoring Division (GMD) and is one of six atmospheric baseline observatories located in remote areas around the world^4^. The other observatories are located at the South Pole, Antarctica; Barrow, Alaska; Cape Matatula, America Samoa; Trinidad Head, California; and Summit, Greenland. In addition to full meteorological measurements, these sites also provide a host of additional measurements including greenhouse gas concentrations. Hourly measurements of climate phenomena are available for all variables and 1-minute data for downward solar radiation are also available. MLO is also critically important for measurements of atmospheric optical depth^19^, ozone^20^, and for instrument calibrations and comparisons e.g. refs 21,22. Data can be accessed directly from the MLO observatory website (https://www.esrl.noaa.gov/gmd/obop/mlo/) and some variables are available through the NCEI electronic repository (https://www.ncei.noaa.gov/).

#### RAWS—National Network

Remote Automated Weather Stations (RAWS) were established and are maintained by the National Interagency Fire Center. RAWS stations are located across the US, the US territories of Puerto Rico and St. Croix in the Caribbean, and on several islands in the Pacific. Most RAWS stations record WS and WD, wind gusts, RF, T_a_, Sw, RH, fuel moisture, atmospheric pressure, SM and T_s_, and measurements are taken at an hourly interval. All instrumentation at each site is situated on a 3 m towers. RF, Ta, Sw and RH are measured at 2 m and WS is measured at 3 m. In Hawai‘i we have identified 64 RAWS stations that had operated for at least one year; 49 stations are currently active in 2017. Daily measurements from this network are available through the RAWS electronic repository (https://raws.dri.edu/wraws/hiF.html). Hourly data are managed by the WRCC and can be obtained by contacting WRCC directly with a request (https://wrcc.dri.edu/). RAWS data obtained directly from the WRCC are not given in SI/Metric units. RF is given in inches (in), Ta in degrees Fahrenheit (˚F), Sw is given in Langleys (˚ly), and wind speed in miles per hour (mph). The RAWS network is part of the GHCN and daily rainfall and temperature data for a select number of stations are available through the NCEI electronic repository (https://www.ncei.noaa.gov/). RAWS data obtained through the NCEI have been converted to SI/Metric.

#### NREL—National Network

The National Renewable Energy Laboratory (NREL) Measurement and Instrumentation Data Center (MIDC), provides irradiance and meteorological data from stations located across the US and US territories in the Caribbean. In Hawai‘i, NREL has operated three climate stations. However, only one of these stations (Kailua-Kona, Hawai‘i ) is currently active. Less than two years of data are available at the discontinued sites on O‘ahu (Kalaeloa) and Lāna‘i (La Ola). Measurements at these locations include hourly RF, WS, atmospheric pressure and RH. One-minute observations of global, direct normal, and diffuse horizontal radiation, are also available at each site. This data can be obtained directly from the MIDC web portal (https://midcdmz.nrel.gov/).

#### USCRN—National Network

Hawai‘i has two stations from NOAA's premiere surface monitoring network, the U.S. Climate Reference Network (USCRN)^4^. Three independent measurements of Ta and RF are made at each station, ensuring continuity of record and maintenance of well-calibrated and highly accurate observations. The stations are placed in pristine environments expected to be free of development for many decades. Stations are monitored and maintained to high standards and are calibrated on an annual basis. In addition to T_a_ and RF, these stations also measure Sw, surface skin temperature, and WS. They also include triplicate measurements of SM and soil temperature at five depths, as well as measurements of RH. Measurements for the two USCRN stations located in Hawai‘i (Hilo and Mauna Loa) began in September of 2005. Hourly data are available at these locations. The USCRN network is part of the GHCN and some of USCRN data including temperature and precipitation can be accessed through the NCEI (https://www.ncei.noaa.gov/). Additional climate variables can be obtained by contacting NCEI directly.

#### SCAN Network—National Network

The Soil Climate Analysis Network (SCAN) program was established by the Natural Resources Conservation Service (NRCS). SCAN stations monitor and report hourly measurements of RF, T_a_, RH, WS, SM, T_s_ and Sw over 200 sites across the U.S. We have identified 8 SCAN stations in Hawai‘i all of which are located on Hawai‘i Island. The Hawai‘i located SCAN stations have been operating since 2005 and all 8 stations were determined to be active at the start of 2017. SCAN data can be downloaded from the SCAN data web portal. (https://www.wcc.nrcs.usda.gov/scan/).

#### ASOS_AWOS—National Network

The Automated Surface Observing Systems (ASOS) program is a joint effort of the National Weather Service (NWS), the Federal Aviation Administration (FAA), and the Department of Defense (DOD). In general, most of these stations are located at Airports across the US and US territories in the Caribbean and the Pacific. These systems report at 1-hour intervals. However, 1-minute and 5-minute ASOS data are available for some variables. These systems also report special observations if weather conditions change rapidly and cross aviation operation thresholds. Automated Weather observing Systems (AWOS) are operated and controlled by the FAA. These stations report at 20-minute intervals. Most ASOS and AWOS stations measure RF, T_a_, WS, and RH. Atmospheric sounding data, measurements of the vertical profile of physical atmospheric properties, are available for the ASOS sites located at Hilo and Līhu‘e airports on Hawai‘i Island and Kaua‘i, respectively. We have identified 15 ASOS_AWOS stations in Hawai‘i, eight of which are active in 2017. The ASOS_AWOS network is part of the GHCN and T_a_ and RF data can be accessed through the NCEI database. Atmospheric sounding data can be obtained by contacting the University of Wyoming, Department of Atmospheric Sciences (http://weather.uwyo.edu/upperair/sounding.html) and through NCEI database (https://www.ncei.noaa.gov/).

#### CraterNet—Hawai‘i-only Network

CraterNet consists of 13 weather stations located in Haleakalā crater (a large erosional depression near the summit) as part of an effort to monitor endemic plant and insect species at high elevations. This network began operating in July of 2010 and spans an elevation gradient of 971 m (1927 to 2898 m), encompassing the major geographic range of the current Haleakalā silversword (*Argyroxiphium sandwicense* subsp. *macrocephalum*), an iconic, threatened plant species endemic to the upper portion of Haleakalā. Station locations were chosen based on population density of the silversword and proximity to existing or planned silversword research plots. The six stations established in 2010 are equipped with instrumentation to measure hourly precipitation, air temperature, relative humidity, soil temperature (5 cm depth), and leaf wetness. Two sensors at each station monitor soil moisture in an area of exposed soil and beneath a healthy plant. In 2016, the network was expanded and seven additional stations were installed. These stations measure RF, T_a_, RH, and soil moisture exclusively. CraterNet was established by and is currently maintained by the project PI at the University of Hawai‘i Mānoa Department of Plant and Environmental Protection Sciences (PEPS). CraterNet data are managed jointly by PEPS and the Giambelluca Ecohydrology Laboratory, University of Hawai‘i at Mānoa (https://sites.google.com/a/hawaii.edu/ecohydrology_lab/home).

#### Little HaleNet—Hawai‘i-only Network

In August of 2005, a network of 12 climate stations was established to increase the density of measurements around the mean elevation of the trade wind inversion on Haleakalā. This network extends westward across slope from the remote HaleNet windward transect, and ranges from ~1980 m to ~2315 m in elevation. The network was designed to complement 134 permanent vegetation plots established during 2003-2006 on the northeast slope of Haleakalā^3^. The climate network consists of three elevational transects, each with one station in the alpine grassland and three stations that evenly bracket the upper cloud forest limit (which approximates the forest-shrubland ecotone)^23^. The climate stations are equipped with instrumentation to measure hourly RF (established 2005), SM (established 2007), T_a_ (established 2008), RH (established 2008), photosynthetically active radiation (established 2011) and T_s_ (2005-2008). The Little HaleNet network, until recently managed by the project PI, will be transferred to the stewardship of the US National Park Service Pacific Island Network Inventory and Monitoring Program. These data are presently managed by the Giambelluca Ecohydrology, University of Hawai‘i at Mānoa (https://sites.google.com/a/hawaii.edu/ecohydrology_lab/home).

#### COOP—International Network

The National Weather Service Cooperative Observer Program (COOP) is an international program that comprises more than 10,000 volunteers who take observations on farms, in urban areas, suburban areas, National Parks, seashores and mountain tops in the US and at least 28 other non-US territories. Meteorological data primarily consists of daily RF, although some stations also report maximum and minimum T_a_. Measurements of RF at 15-minute and 1-hour, intervals are available at select stations. To date, 388 COOP stations have operated in the State of Hawai‘i, and 133 were active at the start of 2017. This network is part of the GHCN and data from most of these stations can be obtained through the NCEI database (https://www.ncei.noaa.gov/). Paper records from many of these stations can also be obtained through the office of the State Climatologist at the University of Hawai‘i at Mānoa (http://www.soest.hawaii.edu/MET/Hsco/scm.html). Some COOP network stations are associated with two or more identification numbers. This is a result of station relocation or large gap in the data record in which a new station is erected in a location where a discontinued station had previously existed. In both cases a unique COOP network ID number is associated with the location of each individual station through time.

#### CoCoRaHs—International Network

The Community Collaborative Rain, Hail & Snow Network (CoCoRaHs) is a grassroots volunteer network of backyard weather observers working together to measure and map precipitation (rain, hail and snow) in their local communities. This network consists of stations located in the United States, Canada, and the Bahamas. We have identified 72 CoCoRaHs stations listed in the NCEI database for Hawai‘i, 47 of which were active in 2017. This network is part of the GHCN and data from all of the stations identified can be obtained through the NCEI database (https://www.ncei.noaa.gov/). Paper records from many of these stations can also be obtained through the office of the State Climatologist at the University of Hawai‘i at Mānoa (http://www.soest.hawaii.edu/MET/Hsco/scm.html).

#### USGS—National Network

The United States Geological Survey (USGS), Pacific Island Water Science Center (PIWSC), operates climate and water monitoring stations in the US and Guam. USGS stations typically provide information on surface water, groundwater, water quality, and RF. We have identified 112 daily RF stations that have operated at some point in time in Hawai‘i, and at least 22 were currently operating at the start of 2017. Data from 56 of these stations can be obtained through the USGS National Water Information System: data portal (https://waterdata.usgs.gov/nwis/sw). Station records not listed on this portal can be obtained as a paper record through the USGS Pacific Island Water Science center (https://hi.water.usgs.gov/) or the office of the State Climatologist at the University of Hawai‘i at Mānoa (http://www.soest.hawaii.edu/MET/Hsco/scm.html).

#### HC&S—Hawai‘i-only Network

The Hawai‘i Commercial and Sugar (HC&S) operated at least 100 RF stations in central Maui between 1931 and 2016. All HC&S data were obtained in inches per day and then converted to millimeters per day. In 2016, HC&S discontinued its operation and climate monitoring efforts. Daily RF data for this network (beyond what is provided in the accompanying data sets) might be difficult to obtain considering HC&S operations have shut down, however, some data might still exist. In addition to the daily RF data, we have identified seven HC&S stations with hourly measurements of RF, T_a_, RH, and Sw and 2 stations with one year of eddy covariance measurements of carbon and water vapor exchange. We have not included these stations in the master list, however, the hourly data have been archived by the Giambelluca Ecohydrology Laboratory located in the Department of Geography at the University of Hawai‘i at Mānoa (https://sites.google.com/a/hawaii.edu/ecohydrology_lab/home).

#### Hydronet—Hawai‘i-only Network

The Hydrological Network of Hawai‘i (HydroNet) system began archiving 15-minute RF data in July 1994. We have identified 69 stations in Hawai‘i and as of 2017, 66 stations were determined to be operational. At least 18 of these stations have been co-located with COOP stations and therefore share State specific identification numbers. Hydronet data are managed by NOAA NWS and updated monthly. The geographical coordinates provided in the datasets accompanying this manuscript have been truncated to the nearest 100th of a degree decimal at the request of the NWS data managers. For a list of more precise coordinates please contact the NWS Pacific Region Headquarters. Hydronet data can be obtained directly through the NWS Pacific Region online data portal (http://www.prh.noaa.gov/hnl/hydro/hydronet/hydronet-data.php).

#### State Network—Hawai‘i-only Network

In addition to the climate stations previously mentioned we have identified 1554 stations have been identified that: 1) do not belong to any of the networks mentioned above, 2) measure RF only, 3) are observed by a local, county, or state entity. For simplicity, we have grouped all of these stations into the “State Network” and provide information about individual station observers in the “Climate_Station_List.csv” accompanying this manuscript. Many of the stations in this State Network are associated with the pineapple and sugar plantation agriculture in Hawai‘i and most of these data are archived in paper format in the Hawai‘i State Climate Office. Other stations have been monitored by local, county, and state agencies, This network also includes 29 stations where vegetation was used as Proxy for estimating mean annual rainfall and 11 stations where rainfall was simulated to improve spatial interpolation for the RF Atlas of Hawai‘i^1^. Giambellluca et al.^1^ obtained many of station records for the State Network and produced a quality-controlled time series of monthly (1920-2012) rainfall which is publicly available via the Rainfall Atlas of Hawai‘i website (http://rainfall.geography.hawaii.edu/).

Only eleven stations within this network are known to be active. Data from active or recently discontinued stations can be obtained by directly contacting the data observer listed in the “Climate_Station_List.csv”. For example, East Maui Irrigation (EMI) currently archives monthly rainfall data from 11 stations, four of which are listed in State Network and nine of which are listed in the COOP network. Currently, only two of these stations report RF to the NCEI. Recent historical EMI data is available in an electronic format and can be obtained by contacting the EMI data manager.

#### The Hawai‘i State Key Number System

Each of the climate monitoring networks described above employs a different numbering and naming system for its stations within their respective networks. The Hawai‘i State Key Number (SKN) system was established in 1948 in joint collaboration between the Hawaiian Sugar Planters’ Association and the Pineapple Research Institute^24^. SKN ranges were assigned to each island and, within each island (Table 3), SKNs are regionally coherent, i.e., stations in close proximity have similar SKNs. Since the system was initiated, several updates have been made to assign SKNs to newly established stations^1,24,25^. In this study, we assigned SKNs to 94 stations that were not previously listed in the system using the following approach. First, all stations with SKNs were plotted using ArcGIS 10.3^17^. Stations without an SKN were also plotted and the ranges of SKN numbers surrounding an unnumbered station were recorded to determine and assign an appropriate new (unused) number. Each of the seven main Hawaiian Islands is associated with a defined range of SKNs (Table 4). Careful attention was given to assigning numbers based on the SKNs of nearby stations and the affiliation of an individual station within a particular network.

In addition to the Hawai‘i SKN, we also provide the NCEI station identification number for all of the stations included in the GHCN. The NCEI climate data online search tool (https://www.ncdc.noaa.gov/cdo-web/search) can be used to access station metadata including: data inventory, available data types, general history, equipment history, and documentation.

### Technical Validation

**Figure 3:**
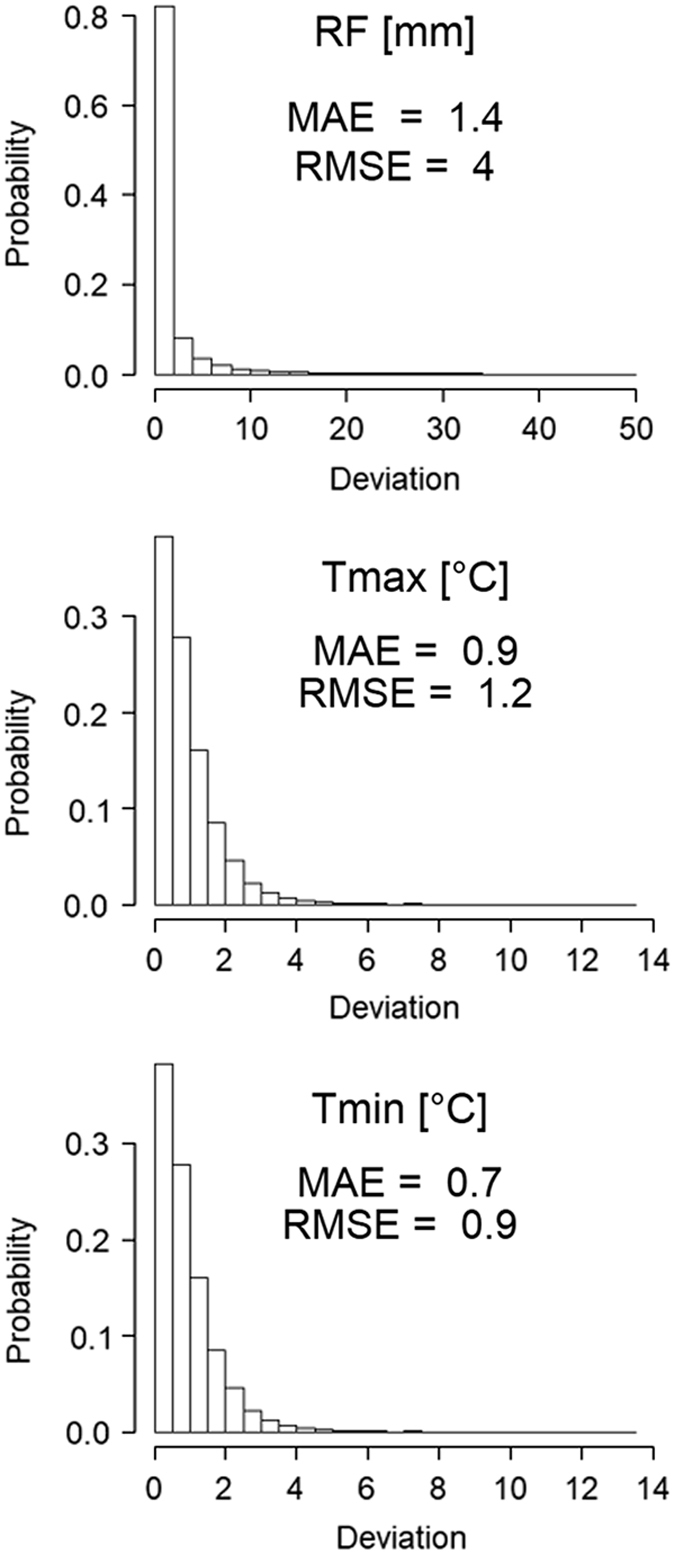
Frequency distributions of absolute errors for gap filled daily data. Rainfall (top pane), maximum temperature (middle pane), and minimum temperature (bottom pane). MAE is mean absolute error; RMSE is root mean square error.

**Figure 4:**
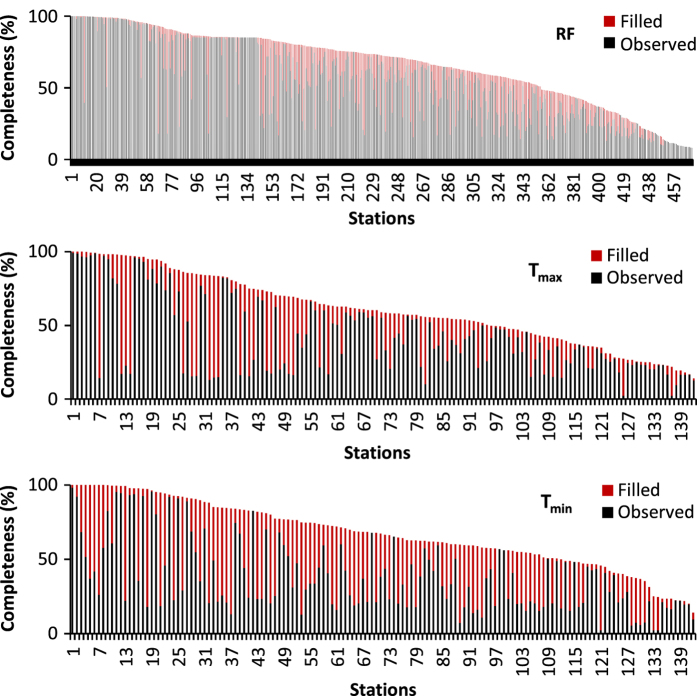
Completeness of station records for daily rainfall and temperature variables. Showing (gray) observed data and (red) gap-filled data for rainfall (top pane), maximum temperature (middle pane) and minimum temperature (bottom pane). Stations are listed in order of data completeness after gap filling was applied.

**Figure 5:**
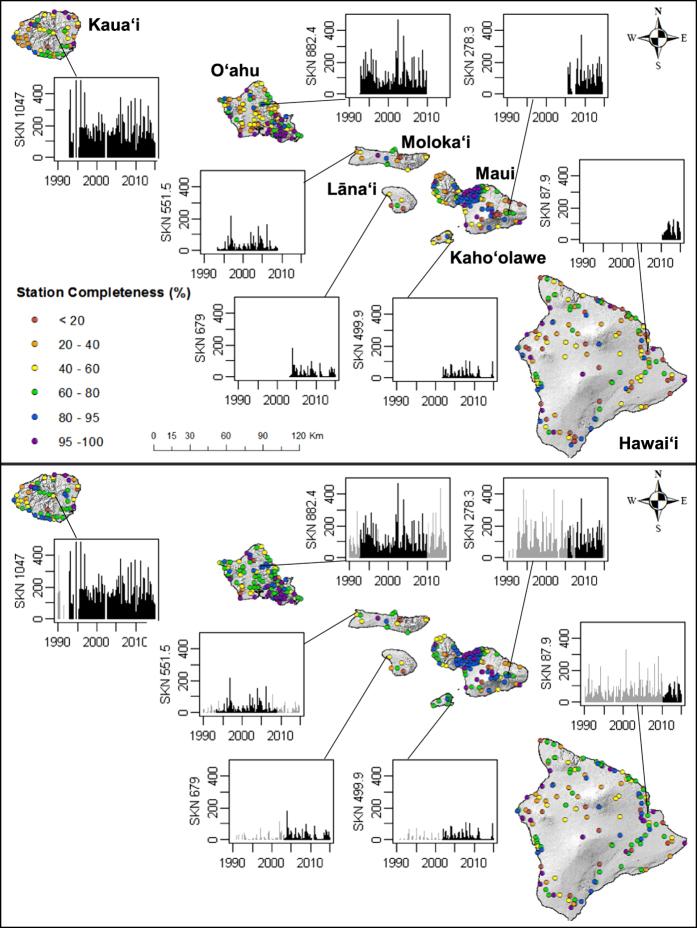
Completeness of rainfall records at 471 raingauge stations in Hawai‘i, before and after gap filling is applied. Station completeness before and after gap filing are shown in the top and bottom panes respectively. Example time series are shown for the same select stations in each pane with black bars representing observed rainfall (mm d^−1^) and grey bars representing filled rainfall.

**Figure 6:**
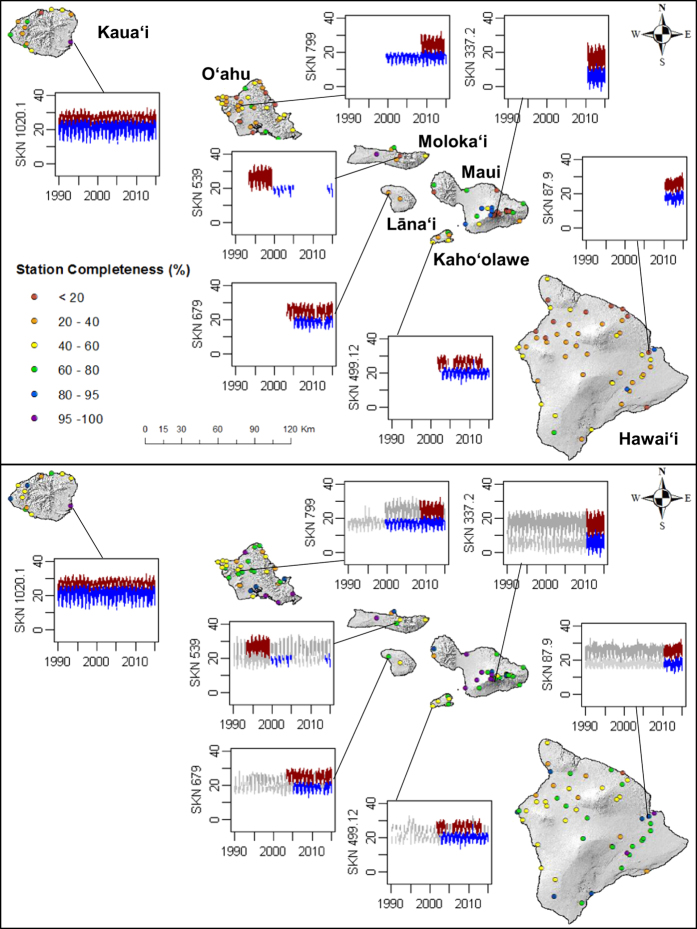
Completeness of temperature records at 142 climate stations in Hawai‘i, before and after gap filling is applied. Station completeness before and after gap filing are shown in the top and bottom panes respectively. Example time series are shown for the same select stations in each pane with red and blue lines representing observed maximum and minimum surface air temperature (°C) respectively and dark gray and light gray lines representing filled maximum and minimum surface air temperature respectively.

Quality control was performed to ensure that gap filling methods (see methods section) used to partially fill RF, T_max_ and T_min_ datasets produce results consistent with the original data for each station. To accomplish this, the unique gap filling methods were used to estimate RF, and T_max_ and T_min_ on days when observations existed. This was done to determine the uncertainty of the gap-filled data by testing them against the actual observations. In total, over 1 million RF and 200,000 T_max_ and T_min_ observations were recreated using the respective gap filling methods for each variable and compared against the actual observations. Mean bias error (MBE), mean absolute error (MAE) and root mean square error (RMSE) statistics^26^ were generated for each dataset (Table 4). The average MBE was low for all three variables indicating that no consistent over- or under- prediction occurred in the gap filled data. Gap filling added 12.7% to the rainfall data set. The mean (standard deviation) of MAE at individual stations was 1.7 (±1.3) mm d^−1^ and of RMSE was 3.9 (±2.1) mm d^−1^. Linear regression gap filling techniques added an additional 18.4 and 29% to the T_max_ and T_min_ datasets, respectively. MAE was 0.9 (±0.2) and 0.7 (±0.2) °C and RMSE was 1.1 (±0.3) and 0.9 (±0.3) for T_max_ and T_min_ respectively. The frequency distribution of the absolute errors are shown in Figure 3. The completeness of the observed and filled datasets is shown for each variable in Figure 4. For RF, 13% of the stations contain data covering at least 95% of the study period. For temperature, 11% of the stations have records that are at least 95% complete. The dataset completeness statistics are given in Table 5. The completeness record at individual stations before and after the gap fill, as well as example station time series is shown for RF (Figure 5) and temperature (Figure 6). Time series were selected to show a range of rainfall and temperature measurements across the Island chain. One station from each of the seven islands was selected.

### Usage Notes

The datasets provided here can be used for a number of applications including the development of gridded climate products (see, for example Di Luzio et al.^27^) at various temporal resolutions, the identification of extreme climatic events and inter-annual variability analyses. In addition, this current endeavour sets a foundation for future updates of the variable time series as data become available. Data provided here might also be combined with various sources of other scientific or social scientific historical time series and or perspectives. Allan et al.^28^ has highlighted the need to incorporate social understandings of climate variability and change with the implications for human vulnerability and resilience. Integrated efforts that combine physical and social science perspectives would provide enhanced climate products and perspectives that could aid policy makers involved in adaptation and management decisions across various scales^28^.

## Additional information

**How to cite this article:** Longman, R. J. *et al.* Compilation of climate data from heterogeneous networks across the Hawaiian Islands. *Sci. Data* 5:180012 doi: 10.1038/sdata.2018.12 (2018).

**Publisher’s note:** Springer Nature remains neutral with regard to jurisdictional claims in published maps and institutional affiliations.

## Supplementary Material



## Figures and Tables

**Table 1:** Climate networks in Hawai‘i.

**Network**	**Spatial-Extent**	**All Sta.**	**Active Sta.**	**Dataset Sta.**	**Observations**	**Meas. Interval**	**First Yr.**	**Last Yr.**
HaleNet	Hawai‘i-only	11	9	11	RF,Ta,RH,WS,Sw,Lw	Hourly	1988	Present
HECO	Hawai‘i-only	8	7	8	RF,Ta,RH,WS,Sw,Lw	10 min	2009	Present
HavoNet	Hawai‘i-only	2	2	2	RF,Ta,RH,WS,Sw,Lw	30 min	2005	Present
ESRL/GMD	International	1	1	1	RF,Ta,RH,WS,Sw,Lw	Hourly (1 min Sw)	1905	Present
RAWS	National	64	56	58	RF,Ta,RH,WS,Sw	Hourly	1990	Present
NREL	National	3	1	1	RF,Ta,RH,WS,Sw	Hourly (1 min Sw)	2010	Present
USCRN	National	2	2	2	RF,Ta,RH,WS,Sw	5 min	2005	Present
SCAN	National	8	8	0	RF,Ta,RH,WS,Sw	Hourly	2005	Present
ASOS_AWOS	National	15	9	8	RF,Ta,RH,WS	Hourly / 1 & 5 min	1905	Present
CraterNet	Hawai‘i-only	13	13	6	RF,Ta,RH	10 min	2010	Present
Little HaleNet	Hawai‘i-only	12	12	12	RF,Ta,RH	Hourly	2005	Present
COOP	International	388	130	196	RF,Ta	Daily/Hourly	1905	Present
CoCoRaHS	International	72	47	10	RF	Daily	1949	Present
USGS	National	112	22	47	RF	Daily	1910	Present
HC&S	Hawai‘i-only	60	0	42	RF	Daily	1910	2016
HydroNet	Hawai‘i-only	69	66	64	RF	15 min	1994	Present
State	Hawai‘i-only	1554	7	0	RF	Monthly/Daily	1838	Present
Where; Network is the measurement network; Spatial-Extent is the geographical extent of the measurement network; All Sta. is the number of stations identified within a given network; Active Sta. is the number of stations active as of 1 January 2017, Observations is the meteorological observations taken within a given network (RF=rainfall; Ta=Surface air temperature; RH=Relative Humidity; WS=Wind speed; Sw=incoming short wave solar radiation; Lw=downwelling longwave radiation): Meas. Interval is the measurement Interval used by each network; First Yr. is the first year of measurements; Last Yr. is the last year of measurements.								

**Table 2:** Description of datasets accompanying this manuscript.

**Data Type**	**SI Units**	**Data Network**	**n Sta**	**Data File**
List of Climate Stations	NA	HaleNet, HECO, HavoNet, ESRL_GMD, RAWS, NREL, ASOS_AWOS, CraterNet, LittleHaleNet, COOP, CoCoRaHs, USGS, HC&S, Hydronet,State	2394	Climate_Station_List.xlsx
Rainfall	mm	HaleNet, HECO, HavoNet, ESRL_GMD, RAWS, NREL, ASOS_AWOS, CraterNet, LittleHaleNet, COOP, CoCoRaHs, USGS, HC&S, Hydronet	471	RF_Data_Not_Filled.txt RF_Data_Filled.txt
Maximum Temperature	°C	HaleNet, HECO, HavoNet, ESRL_GMD, RAWS, NREL, ASOS_AWOS, CraterNet, LittleHaleNet, COOP, CoCoRaHs	142	Tmax_Data_Not_Filled.txt Tmax_Data_Filled.txt
Minimum Temperature	°C	HaleNet, HECO, HavoNet, ESRL_GMD, RAWS, NREL, ASOS_AWOS, CraterNet, LittleHaleNet, COOP, CoCoRaHs	142	Tmin_Data_Not_Filled.txt Tmin_Data_Filled.txt
Relative Humidity	%	HaleNet, HECO, HavoNet, ESRL_GMD, RAWS, CraterNet, LittleHaleNet	105	RH_DataFile.txt
Wind Speed	m/s	HaleNet, HECO, HavoNet, ESRL_GMD, RAWS, NREL	87	WS_DataFile.txt
Incoming Shortwave solar radiation	W m^−2^	HaleNet, HECO, HavoNet, ESRL_GMD, RAWS, NREL	82	Sw_DataFile.txt
Downwelling Longwave radiation	W m^−2^	HaleNet, HECO, HavoNet, ESRL_GMD	18	Lw_DataFile.txt
Where: Data Type is the variable or information included in the dataset; SI Units is scientific units of the variable expressed in the dataset; Data Network shows the individual networks that data was drawn from; n Sta. is the number of stations used in the dataset; Data File is the name of the dataset file.				

**Table 3:** State Key Number (SKN) system number range for each Hawaiian Island.

**Island**	**SKN range**
Hawai‘i	1–235
Maui	236–497
Kaho‘olawe	498–499
Moloka‘i	500–599
Lāna‘i	600–699
O‘ahu	700–924
Kaua‘i	925–1149
Niihau	1150
Where SKN range is the range of State key numbers used on each respective island.	

**Table 4:** Gap filling statistics for rainfall, maximum temperature and minimum temperature.

**Gap-Fill Errors**	**Rainfall (mm)**	**T**_**max**_ **(°C)**	**T**_**min**_ **(°C)**
MBE±sd	0.0±0.2	0.0±0.0	0.0±0.0
MAE±sd	1.4±1.3	0.9±0.2	0.7±0.2
RMSE±sd	4±2.1	1.2±0.3	0.9±0.3
Where; MBE is mean bias error; MAE is mean absolute error; RMSE is root mean square error; sd is the standard deviation of the error. Error statistics are given as the average of all stations.			

**Table 5:** Dataset completeness before and after gap filling techniques were applied for rainfall, maximum temperature and minimum temperature.

**Data Completeness**	**Rainfall (%)**	**Tmax (%)**	**Tmin (%)**
Observed Dataset	53.9	42.4	38
Partially Filled Dataset	66.6	60.8	67
Where: Observed Dataset is the completeness of the obaservational datqaset before gap filling (1990-2014); Partially Filled Dataset is the completeness of the gap-filled dataset (1990-2014).			

## References

[d1] Figshare Clark M. P. 2018 http://doi.org/10.6084/m9.figshare.c.3858208

